# Can alexithymia be assessed through an interview in adolescents? The Toronto Structured Interview for Alexithymia: Reliability, concurrent validity, discriminant validity, and relationships with emotional-behavioral symptoms

**DOI:** 10.3389/fpsyt.2022.1055946

**Published:** 2023-01-18

**Authors:** Stefania Muzi, Michela Di Trani, Alessia Renzi, Cecilia Serena Pace

**Affiliations:** ^1^Department of Education Sciences, School of Social Sciences, University of Genoa, Genoa, Italy; ^2^Department of Dynamic, Clinical Psychology and Health, Faculty of Medicine and Psychology, Sapienza University of Rome, Rome, Lazio, Italy

**Keywords:** TSIA, 20-item Toronto Alexithymia Scale, adolescents, alexithymia, psychometric proprieties, internalizing and externalizing disorders, clinical adolescent sample, community adolescents

## Abstract

Alexithymia is connected to adolescents' psychopathology, but the current methods of assessment present limitations. The Toronto Structured Interview for Alexithymia (TSIA) was developed to overcome the limits of the main used self-rating scale in adults, but no studies investigated its feasibility with adolescents. This study involved 95 community adolescents aged 12–19 years. Adolescents were assessed with the TSIA, the 20-item Toronto Alexithymia Scale (TAS-20), the Verbal Comprehension Index of the WISC-IV for verbal skills, and the Child Behavior Checklist and Youth Self Report for emotional-behavioral symptoms. The aims were to investigate the TSIA internal consistency, concurrent validity with the TAS-20, discriminant validity with participants' verbal skills, and relationships with emotional-behavioral symptoms. TSIA showed good internal consistency, concurrent validity with the TAS-20 (except for factor DDF), and independence by participants' verbal skills, but few relationships with emotional-behavioral symptoms. In conclusion, TSIA showed some good psychometric proprieties but little convergence with research findings obtained with the TAS-20, suggesting the need for further research to check the feasibility of using the TSIA with adolescents. Meanwhile, a precautionary multi-method assessment of alexithymia is recommended.

## 1. Introduction

Alexithymia is defined as a personality trait characterized by difficulties in identifying and verbally describing bodily sensations and emotions, together with a concrete cognitive style and lack of fantasy (1). The scientific community proposed a developmental theory of the origin of alexithymia (2), whereby signs of poor emotional awareness and difficulties in verbalizing emotions in adolescents can predispose them to develop alexithymia as a stable personality trait in adulthood (3–6). Moreover, the assessment of alexithymia in adolescents raises interest because higher levels of alexithymia in youths are associated with more internalizing and externalizing symptoms and poor physical health (4, 7–12). However, researchers are debating whether to assess alexithymia in adolescents by self-rated questionnaires or observer-rated methods (13, 14). At present, almost all published studies with adolescents have employed the 20-item Toronto Alexithymia Scale [TAS-20; (1)], a self-report questionnaire that assesses alexithymia as a multidimensional construct composed of three factors—namely (1) Difficulty Identifying Feelings (DIF), (2) Difficulty Describing Feelings (DDF) and (3) Externally Oriented Thinking (EOT)—of which the sum corresponds to a total score of alexithymia. However, the use of TAS-20 with adolescents reveals numerous limits (14–16). For instance, Parker et al. (17) report fluctuations of alexithymia in different age groups, highlighting the difficulty of capturing alexithymia during adolescence when emotion regulation abilities are still under development. Moreover, variables such as the level of education can influence reading comprehension (18), and adolescents may find it more difficult to understand the questions (19), with no researchers who could provide additional inquiring and explanations.

To overcome these limits, some authors (20, 21) propose to assess adolescents' alexithymia through a multi-method approach including both the TAS-20 and the clinician-rated Toronto Structured Interview for Alexithymia [TSIA; (22)], suggesting the latter as more sensitive to capture alexithymia in adolescents. The TSIA has been developed based on the TAS-20 and by the same authors, who detailed the measure development and psychometrics in Bagby et al. (22). This interview counts 24 questions, six for each dimension assessed, i.e., the three TAS-20 factors DIF, DDF, and EOT, plus a fourth additional factor Imaginative Processes (IMP), and the two macro-factors Affective Awareness (AA; the sum of DIF and DDF) and Operative Thinking (OT; the sum of EOT and IMP). The reason TSIA could overcome the limitations of TAS-20 with adolescents is that each TSIA question is followed by in-depth questions and requests for examples, solving misunderstandings. Moreover, an expert clinician administers TSIA and rates responses after having attended specific and mandatory training. Furthermore, the TSIA can be audio-recorded and transcribed to allow other trained experts to check assigned scores, also evaluating the accordance between different raters.

The TSIA has been mainly used with clinical and community adults, showing good psychometric properties in terms of internal consistency, inter-rater reliability, construct validity, language invariance, and concurrent validity with the TAS-20 (21–29). However, the evidence of TSIA psychometric proprieties with adolescents is limited to two studies. Specifically, Montebarocci and Surcinelli (27) involved a subgroup of community adolescents aged 17–19 years within a larger sample of adults, reporting good TAS-20-TSIA accordance but without reporting isolated data of this subgroup. Balottin et al. (20) involved a clinical group of girls aged 13–17 years, finding that the TAS-20 and TSIA led to similar results concerning DIF and DDF, showing disagreement in assessing total alexithymia and the factor EOT. However, none of these studies explored the independence of results from participants' verbal skills, which can influence participants' comprehension and responses (19). Moreover, despite age differences through the use of the TAS-20 in adolescents emerged (17) no studies investigating this issue through the TSIA were found. As a consequence, there is no information on the relationship between alexithymia measured through the TSIA and adolescent emotional-behavioral symptoms to support research findings obtained using the TAS-20 and indicating alexithymia as a transdiagnostic risk factor for adolescent mental health (12).

### 1.1. The current study

Given the literature gaps stated above, the general aim of this study was to investigate the psychometric properties of the Toronto Structured Interview for Alexithymia in non-clinical community adolescents.

In a sample of community adolescents, checking for gender, age, and years of school, the specific aims of the study were to investigate:

(a) The internal reliability of the TSIA, expecting Cronbach's alphas > 0.60 as in Montebarocci and Surcinelli (27).
(b) The concurrent validity of the TSIA with the TAS-20, expecting correlations in total and paired factors scores DIF, DDF, EOT.
(c) The discriminant validity of the TSIA to participants' verbal skills.
(d) The associations between TSIA with emotional-behavioral symptoms. Positive correlations between higher total and factor scores of alexithymia and more internalizing and externalizing symptoms were expected based on studies with the TAS-20.

## 2. Method

### 2.1. Participants and procedure

Recruitment took place between 2018 and 2020 through Ligurian public schools, within larger research that obtained ethical approval by the Department Ethical Committee of the University of Genoa (Italy), protocol no. 021, and Social and Health Services with protocol no. PG/2017/368220. Inclusion criteria were age between 12 and 19 years old and an absence of diagnosis for severe physical or intellectual disabilities.

With the cooperation of the school principals, the adolescents and their legal caretakers were informed of the research purposes and procedures. They were provided with an informed consent written according to the declaration of Helsinki, which adolescents and parents of minors have to sign to agree to the adolescent's voluntary participation in the research. Participants did not receive any incentive for their participation, and they were ensured that they could drop or not fill part of the measures at any time, without justification.

All one-hundred-one community adolescents contacted agreed to participate in the entire research. The current study only includes participants completing both TSIA and TAS-20 without missing items, so participants in this study were 95 non-clinical community adolescents, of whom Table 1 displays full details.

**Table 1:** Sociodemographic characteristics of 95 community adolescents.

	***n***	**%**	***M***	**SD**
Age (years)	95		15.67	2.07
**Gender**				
Male	43	45.3		
Female	52	54.7		
**Education**				
Middle school	71	74.7		
High school	21	22.1		
School ended	3	3.2		
**Parents** ^a^				
Together	69	73.4		
Not together	19	20.2		
Single	5	5.3		
Widowed	1	1.1		
Have a job	176	98.3		
Years of education			13.3	3.84
**Siblings** ^b^				
No	19	20		
1	52	54.7		
2 or more	17	19.3		

^a^Counted on 179 parents (11 missing).

^b^Counted on 88 participants.

The data collection took place in University laboratories, where MSc students in clinical psychology trained by the authors administered the TSIA and a test for verbal skills to each adolescent, who later filled autonomously the TAS-20 and a questionnaire for emotional-behavioral problems (see Section 2.2). The entire session lasted around 1.5 h. Meanwhile, in another room, the parent who spent more time with the adolescent (94% mothers) filled out the parent version of the same questionnaire for emotional-behavioral problems, the Child Behavior Check List 6–18 years (see Section 2.2).

According to TSIA scoring guidelines, the MSc students assigned scores during the interview, but the TSIAs were audiotaped and verbatim transcribed to allow supervision and scores' correction by the authors' expert in the TSIA, who assigned final scores. The first author supervised and rated the transcriptions of all TSIA, and fifteen of them were also double-coded by the third author (17%). The two raters reached 98% of the agreement.

Measures for alexithymia and emotional-behavioral problems were collected for all participants, while the test for verbal skills was completed by 52 participants (45.26% attrition), as it was not administered to participants aged 17–19 (*n* = 43) because the test suitability, see below.

### 2.2. Measures

The original 24-question version of the Toronto Structured Interview for Alexithymia [TSIA; (22)] is described above. Each question can be rated from 0 to 2, with scores in each dimension (DIF, DDF, EOT, IMP, AA, OT) ranging from 0 to 12, the total score ranging from 0 to 48, and higher scores indicating more alexithymia. The translation here used showed Cronbach's α 0.86 for the total score, ranging from 0.70 (EOT) to 0.82 (AA) for the dimensions (23).

The 20-items Toronto Alexithymia Scale [TAS-20; (1, 30)] is the 20-item self-report questionnaire described in the introduction. This questionnaire provides scores in DIF, DDF, and EOT factors, summed in a total score of alexithymia ranging from 20 to 100. Cronbach's α for internal reliability of the total score were 0.75 and 0.82 for the version used in this study. Alphas for this version ranged between 0.52 (EOT) to 0.77 (DIF) in community adults.

The Verbal Comprehension Index of the Weschler Intelligence Scale [VCI-WISC-IV; (19, 31)] was used to assess the verbal skills of participants aged 12–16 years and 11 months. This index is the sum of the weighted scores of the three subtests Vocabulary, Similarities, and Comprehension. Cronbach's α for the Italian version here used is 0.96, ranging from 0.69 for comprehension to 0.94 for vocabulary.

The Child Behavior Check List 6–18 and the Youth Self-Report 11–18 years [CBCL and YSR; (32, 33)] are two questionnaires used to assess emotional-behavioral symptoms of the adolescent in the past 6 months, as rated by a parent in the CBCL and by the adolescent in the YSR. They provide scores in eight syndromes scales, returning three main scores of Internalizing problems (including anxious/depressed, withdrawn/depressed, somatic complaints), Externalizing problems (aggressive and rule-breaking/delinquent behaviors), and a score of total problems, which counts also scores in other syndrome scales (i.e., social problems, attentional problems, thought problems). In the Italian versions here used, Cronbach alphas were all > 0.60.

A socio-demographic sheet *ad hoc* collected information about participants and their families.

### 2.3. Data analyses

All analyses were considered significant with *p* < 0.05. Cronbach's alphas were computed to check the internal consistency of the TSIA. *T*-test for independent samples was used to preliminary check gender differences. Pearson's r correlation coefficient was employed to investigate TSIA associations with participants' age, years of education, its convergent validity with the TAS-20, the discriminant validity concerning participants' verbal skills, and associations with CBCL 6–18 and YSR 11–18.

## 3. Results

Preliminary analyses revealed no gender differences in TSIA scores (all *p* > 0.950, see Supplementary Table). The total (*r* = −0.314, *p* = 0.002) and EOT (*r* = −0.243, *p* = 0.018) scores in the TSIA decreased with the age, and these two TSIA factors showed also negative correlations with the years of education, respectively (with total score *r* = −0.501, *p* < 0.001, and EOT, *r* = −0.298, *p* = 0.003). Although the TAS-20 was not the focus of this study, its relations with demographic variables were checked: females showed more DIF and DDF than males (see Supplementary Table). Moreover, total alexithymia (*r* = −0.338, *p* = 0.001) and EOT (*r* = −0.492, *p* < 0.001) of all participants decreased with the age, and EOT also decreased with more years of education (*r* = −0.350, *p* < 0.001).

Table 2 reports descriptive and internal reliability values of all measures. TSIA values were all 0.60 or more, as hypothesized.

**Table 2:** Mean scores, standard deviations, and Cronbach's alphas (α) for all measures^a^ in 95 community adolescents.

	***M***	**SD**	**α**
TSIA total	11.11	7.76	0.88
DIF	3.76	2.35	0.72
DDF	5.21	2.66	0.73
EOT	6.36	2.84	0.73
IMP	5.37	2.83	0.75
AA	11.32	4.78	0.65
OT	18.97	7.57	0.69
TAS-20 total	52.11	10.14	0.80
DIF	16.17	6.05	0.81
DDF	14.74	4.11	0.76
EOT	21.20	4.86	0.50
WISC-IV VCI	98.81	19.39	0.60
YSR total	66.97	20.42	0.88
Internalizing	16.26	9.40	0.82
Externalizing	11.52	6.17	0.85
CBCL total	26.70	17.28	0.90
Internalizing	9.86	6.33	0.81
Externalizing	6.72	6.14	0.88

^a^TSIA, Toronto Structured Interview for Alexithymia; DIF, Difficulty Identifying Feelings; DDF, Difficulty Describing Feelings; EOT, Externally Oriented Thinking; IMP, lack of Imaginative Processes; AA, Affective Awareness; OT, Operative Thinking; TAS-20, Toronto Alexithymia Scale 20 items; WISC-IV VCI, Verbal Comprehension Index of the Weschler Intelligence Scale for Children – IV edition; YSR, Youth Self Report 11–18 years; CBCL, Child Behavior Check List 6–18 years.

Table 3 shows the correlations between the TSIA and TAS-20 which mainly confirmed the hypotheses, except for the absence of an association between TSIA and TAS-20 DDF factor.

**Table 3:** Correlations between the Toronto Structured Interview for Alexithymia^a^ and the Toronto Alexithymia Scale 20 items^b^ in 95 non-clinical adolescents.

**Variable^c^**	**1**	**2**	**3**	**4**	**5**	**6**	**7**	**8**
1. TSIA/total score	—							
2. TSIA/DIF	0.74^***^	—						
3. TSIA/DDF	0.65^***^	0.44^***^	—					
4. TSIA/EOT	0.45^***^	0.37^***^	0.52^***^	—				
5. TAS-20/total score	0.30^**^	0.29^**^	0.26^*^	0.26^*^	—			
6. TAS-20/DIF	0.23^*^	0.37^***^	0.14	0.07	0.81^***^	—		
7. TAS-20/DDF	0.08	0.12	0.11	0.15	0.73^***^	0.50^***^	—	
8. TAS-20/EOT	0.28^**^	0.03	0.29^**^	0.34^**^	0.42^***^	−0.04	0.00	—

Analyses have been controlled for participants' age in years and years of education.

^a^TSIA.

^b^TAS-20.

^c^DIF, Difficulty Identifying Feelings; DDF, Difficulty Describing Feelings; EOT, Externally Oriented Thinking.

^*^p < 0.05, ^**^p < 0.01, ^***^p < 0.001.

Moreover, once ascertained that WISC-VCI scores did not correlate with years of education (*p* = 0.138), there were no significant correlations between TSIA and WISC-VCI scores, all *p* > 0.195, confirming the independence of TSIA results from participants' verbal skills.

Further, Table 4 reports correlations between the two measures of alexithymia and the emotional-behavioral problems as reported by the adolescent (YSR) and by the main caregiver (CBCL). Adolescents who scored higher in the TSIA/DIF reported more total externalizing problems by the YSR, while adolescents showing higher TSIA/total and TSIA/DDF, had caregivers who reported they showed more internalizing problems through the CBCL, and DDF was also related to more total problems.

**Table 4:** Correlations between the alexithymia scores in the Toronto Structured Interview for Alexithymia^a^, the Toronto Alexithymia Scale 20 items^b^ and the emotional-behavioral problems^c^ in 95 community adolescents.

**Variable**	**YSR**			**CBCL**		
	**Total**	**Internalizing**	**Externalizing**	**Total**	**Internalizing**	**Externalizing**
TSIA/total	0.09	0.12	0.03	0.14	0.21^*^	0.07
TSIA/DIF	0.27^*^	0.18	0.23^*^	0.22^*^	0.20	0.17
TSIA/DDF	0.10	0.04	0.12	0.25^*^	0.24^*^	0.19
TSIA/EOT	0.00	−0.10	0.05	0.03	0.11	0.01
TSIA/IMP	−0.13	−0.20	−0.00	−0.11	−0.03	−0.07
TSIA/AA	−0.07	−0.18	0.05	−0.03	0.04	−0.01
TSIA/OT	0.10	−0.05	0.20	0.15	0.14	0.13
TAS-20/total	0.51^**^	0.52^**^	0.31^**^	0.24^*^	0.22*	0.19
TAS-20/DIF	0.62^**^	0.62^**^	0.43^**^	0.22^*^	0.21*	0.15
TAS-20/DDF	0.36^**^	0.36^**^	0.09	0.21^*^	0.20	0.15
TAS-20/EOT	−0.05	−0.04	0.02	0.16	0.01	0.07

Analyses have been controlled for participants' age in years and years of education.

^a^TSIA, Toronto Structured Interview for Alexithymia; DIF, Difficulty Identifying Feelings; DDF, Difficulty Describing Feelings; EOT, Externally Oriented Thinking; IMP, lack of Imaginative Processes; AA, Affective Awareness; OT, Operative Thinking.

^b^TAS-20.

^c^YRS, Youth Self Report 11–18; CBCL, Child Behavior Checklist 6–18 years.

^*^p < 0.05, ^**^p < 0.01.

Differently, TAS-20 total and DIF were related to total and internalizing problems both in YSR and CBCL, and to externalizing problems in the YSR, while factor DDF showed relations with internalizing problems in both YSR and CBCL.

## 4. Discussion

This is the first study reporting psychometric characteristics of the Toronto Structured Interview for Alexithymia (TSIA) in a sample of community adolescents aged from 12 to 19 years.

First, the preliminary control for demographics confirmed the gender invariance of results using the TSIA, as for adults (26), whereas using the TAS-20 girls showed more alexithymia levels—in terms of DIF and DDF—than boys, in accordance with the literature on adolescents (4, 8, 9, 16, 34). This result suggests the need for further clarification to understand if the TSIA could be a good option to overcome eventual gender-response biases in the TAS-20 (17, 20), through the evaluation of an external clinician, or if this result would be attribute to other explanations. However, TSIA results were not independent of participants' age, as younger adolescents showed higher levels of EOT and total alexithymia, in line with the literature with the TAS-20 (17). This may suggest the importance to consider the age of interviewee in case of alexithymia assessment in early adolescents, stressing the need to develop age-adapted measures for youngers, e.g., (7, 35). Moreover, preliminary results indicated lower alexithymia with the TSIA as more education years participants attended. So far, research mainly has focused on the impact of higher emotional awareness—a basic component of alexithymia if low—in increasing academic performance (36). However, some educational aspects can positively intervene in emotional development (37) potentially reducing alexithymia, and they should be further investigated.

Concerning results, Cronbach's alphas revealed good internal reliability of the TSIA in this community sample, in line with the literature on adults (21–23, 25, 27).

Moreover, once controlled for age and years of education, overall results suggested concurrent validity between the interview TSIA and the questionnaire TAS-20 most used with adolescents, in line with literature on adults (22, 23). The only exception was the factor Difficulty Describing Feelings, for which future studies are needed for clarifing the absence of concurrent validity in this factor. Probably one of the two methods can be less sensitive than the other for the use with adolescents. Otherwise, adolescents self-rate their ability to verbally express feelings differently than an expert observer, maybe because their emotional awareness is still under development and they can struggle to provide an evaluation of their ability to describe so intense and fluctuating feelings.

As a novelty, this study first tested and found the independence of TSIA scores from participants' cognitive-verbal abilities, suggesting the discriminant validity of this alexithymia interview from community participants' verbal skills. However, the results of the current study alone do not fill the research gap on the topic, as they cover an age range from 12 to 16 years and 11 months, without providing information about older participants due to a limit of the measure employed. This suggests the need for other studies on the topic to support this result. The years of education were not related to participants' verbal skills and this reinforce the hypothesis of relations between alexithymia and aspects of formal education different than verbal-cognitive skills, which should be further investigated (37).

Lastly, the associations between TSIA scores and adolescents' internalizing and externalizing symptoms were explored, by YSR and CBCL. Only few relationships were found—especially with factor DIF and DDF—therefore results did not fully confirmed the hypotheses based on the literature that employed the TAS-20 with adolescents (25, 38). However, when the TAS-20 was employed, there were several relationships between alexithymia self-rated by adolescents and both self- and parent-reported symptoms, particularly with internalizing problems, in line with the broader literature with this questionnaire (25, 27, 38). These unclear results may suggest an underestimation of the relationships between alexithymia and emotional-behavioral symptoms in adolescents employing the TSIA, or an overestimation employing the TAS-20, which future mixed-method studies should clarify. Maybe relating TSIA and TAS-20 results with a double measure of participants' symptoms -clinician-performed and questionnaire-performed- would help to understand these discrepancies.

Overall, this original study—the first employing the TSIA in a community sample—suggests some psychometric strengths of the TSIA with adolescents, such as good internal validity, concurrent validity with TAS-20 total, DIF and EOT scores, and independence of the results by participants' verbal skills, supporting a reliable use of this interview with underage participants (25). However, findings showed discrepant results with literature in terms of associations with internalizing and externalizing symptoms. In particular these discrepancies call for more research with the TAS-20 as well, to improve the certainty of the current knowledge on connections between alexithymia and community adolescents' mental health through multi-method approach, and plan empirically-driven community prevention in adolescent populations.

Nevertheless, this study has several limitations. First, TSIA and TAS-20 standardized scores were not compared, preventing exploring the reasons for the lack of convergence (4, 17), whereas the concurrent validity with the TAS-20 appears as a crucial issue deserving more investigation. Especially concerning factor EOT, which showed unacceptable reliability in this study as in others with adolescents (15–17). Moreover, the sample is limited and larger samples are needed for further study replications. Additionally, informal observation and comments from participants also converge in suggesting that the original version of the TSIA could be excessively long for adolescents. Future studies can try to use a recently developed shorter version of the TSIA (39), comparing the results with those obtained with the original TSIA, and the TAS-20 as well, to provide professionals with a shorter tool.

In conclusion it is recommended the use a multi-method approach to assess alexithymia in adolescents, until future studies will have address the weaknesses of both TSIA and TAS-20 that emerged from this study.

## Data availability statement

The raw data supporting the conclusions of this article will be made available on request from the corresponding author CSP without undue reservation.

## Ethics statement

The studies involving human participants were reviewed and approved by Department Ethical Committee of the University of Genoa, protocol no. 021. Written informed consent to participate in this study was provided by the participants' legal guardian/next of kin.

## Author contributions

SM co-designed the research and the work, collected and analyzed data, interpreted results, drafted the first version of the work, and critically revised all subsequent drafts for important intellectual content. MDT and AR collected data, interpreted results, and critically revised all drafts and the intellectual contents of the work. CSP co-designed the research and supervise it, interpreted results, and critically revised all subsequent drafts for important intellectual content. All authors add a substantial contribution to the conception and design of the work, or to the acquisition, analysis, or interpretation of data for the work, agree to be accountable for all aspects of the work in ensuring that questions related to the accuracy or integrity of any part of the work are appropriately investigated and resolved, and provided approval for publication of the content.
