# Supplementary material for: Can alexithymia be assessed through an interview in adolescents? The Toronto Structured Interview for Alexithymia: Reliability, concurrent validity, discriminant validity, and relationships with emotional-behavioral symptoms
Source: Front Psychiatry. 2023 Jan 18;13:1055946. doi: 10.3389/fpsyt.2022.1055946 (PMC9889651; doi:10.3389/fpsyt.2022.1055946)
Supplement: Supplementary file 1 [file Table_1.docx]

**Supplementary Table**

*Gender differences in all measures^a^ for two groups of community (n = 95) and clinical (n = 23) adolescents.*

|  |  |  | Community adolescents | | | | | | |  |
| --- | --- | --- | --- | --- | --- | --- | --- | --- | --- | --- |
|  |  |  | Males | |  | Females | |  |  |  |
|  |  |  | *M* | *SD* |  | *M* | *SD* | *t* | *p* |  |
| TSIA total |  |  | 9.88 | 6.27 |  | 12.12 | 8.73 | -1.40 | .164 |  |
| DIF |  |  | 12.12 | 8.73 |  | 3.53 | 2.26 | -0.84 | .402 |  |
| DDF |  |  | 3.53 | 2.26 |  | 3.94 | 2.42 | 0.54 | .593 |  |
| EOT |  |  | 3.94 | 2.42 |  | 5.37 | 2.90 | 0.92 | .362 |  |
| IMP |  |  | 5.37 | 2.90 |  | 5.08 | 2.47 | 0.67 | .507 |  |
| AA |  |  | 5.08 | 2.47 |  | 6.65 | 2.89 | 1.27 | .206 |  |
| OT |  |  | 6.65 | 2.89 |  | 6.12 | 2.79 | 1.69 | .095 |  |
| TAS-20 total |  |  | 50.21 | 9.21 |  | 53.67 | 10.69 | -1.67 | .098 |  |
| DIF |  |  | 13.67 | 10.69 |  | 14.47 | 5.48 | -2.57 | **.012** |  |
| DDF |  |  | 14.47 | 5.48 |  | 17.58 | 6.18 | -2.08 | **.041** |  |
| EOT |  |  | 17.58 | 6.18 |  | 13.79 | 3.60 | 1.38 | .170 |  |
| WISC-IV VCI |  |  | 98.23 | 21.71 |  | 99.38 | 17.17 | -0.21 | .833 |  |
| YSR total |  |  | 64.93 | 22.86 |  | 68.65 | 18.22 | -0.88 | .379 |  |
| internalizing |  |  | 68.65 | 18.22 |  | 12.81 | 8.59 | -3.43 | **.001** |  |
| externalizing |  |  | 12.81 | 8.59 |  | 19.12 | 9.15 | 2.07 | **.042** |  |
| other |  |  | 19.12 | 9.15 |  | 12.93 | 6.82 | 0.18 | .858 |  |
| CBCL total |  |  | 25.77 | 18.97 |  | 10.35 | 5.38 | -0.48 | .633 |  |
| internalizing |  |  | 27.49 | 15.87 |  | 8.67 | 4.34 | -2.21 | **.030** |  |
| externalizing |  |  | 8.33 | 5.90 |  | 8.52 | 4.08 | 0.74 | .464 |  |

^a^TSIA = Toronto Structured Interview for Alexithymia. DIF = Difficulty Identifying Feelings. DDF = Difficulty Describing Feelings. EOT = Externally Oriented Thinking. IMP = lack of Imaginative Processes. AA = Affective Awareness. OT = Operative Thinking; TAS-20 = Toronto Alexithymia Scale 20 items; WISC-IV VCI = Verbal Comprehension Index of the Weschler Intelligence Scale for Children – IV edition; YSR = Youth Self Report 11-18 years; CBCL = Child Behavior Check List 6-18 years.
